# Elucidation of Cellular Responses in Non-human Primates With Chronic Schistosomiasis Followed by Praziquantel Treatment

**DOI:** 10.3389/fcimb.2020.00057

**Published:** 2020-02-24

**Authors:** Michael W. Melkus, Loc Le, Arif J. Siddiqui, Adebayo J. Molehin, Weidong Zhang, Samra Lazarus, Afzal A. Siddiqui

**Affiliations:** ^1^Department of Surgery, School of Medicine, Texas Tech University Health Sciences Center, Lubbock, TX, United States; ^2^Center for Tropical Medicine and Infectious Diseases, Texas Tech University Health Sciences Center, Lubbock, TX, United States; ^3^Department of Internal Medicine, School of Medicine, Texas Tech University Health Sciences Center, Lubbock, TX, United States; ^4^Department of Biology, University of Hail, Hail, Saudi Arabia

**Keywords:** *Schistosoma mansoni*, praziquantel, disease models, *Papio anubis*, cellular immune response, chronic disease, mass drug administration

## Abstract

For decades, mass drug treatment with praziquantel (PZQ) has been utilized to treat schistosomiasis, yet reinfection and the risk of drug resistance are among the various factors precluding successful elimination of schistosomiasis. Tractable models that replicate “real world” field conditions are crucial to effectively evaluate putative schistosomiasis vaccines. Herein, we describe the cellular immune responses and cytokine expression profiles under field conditions that include prior infection with schistosomes followed by treatment with PZQ. Baboons were exposed to *Schistosoma mansoni* cercariae through trickle infection over 5 weeks, allowed for chronic disease to develop, and then treated with PZQ. Peripheral blood mononuclear cells (PBMCs) were monitored for cellular immune response(s) at each disease stage and PZQ therapy. After initial infection and during chronic disease, there was an increase in non-classical monocytes, NK and NKT cells while the CD4:CD8 T cell ratio inverted from a 2:1 to 1:2.5. The cytokine expressions of PBMCs after trickle infections were polarized more toward a Th2 response with a gradual increase in Th1 cytokine expression at chronic disease stage. Following PZQ treatment, with the exception of an increase in B cells, immune cell populations reverted back toward naïve levels; however, expression of almost all Th1, Th2, and Th17 cytokines was significantly increased. This preliminary study is the first to follow the cellular immune response and cytokine expression profiles in a non-human primate model simulating field conditions of schistosomiasis and PZQ therapy, providing a promising reference in predicting the immune response to future vaccines for schistosomiasis.

## Introduction

Parasites have coevolved with their hosts to maintain a tenuous balance between propagation of the parasite life cycle and host immunopathology. Humans have been aware of schistosomiasis since at least 1900 BCE, with hieroglyphic script of hematuria found in the Kahun papyrus and other clinical symptoms in the Ebers and Berlin papyri (Shokeir and Hussein, 1999). Schistosomiasis, a neglected tropical disease, is considered by the World Health Organization to be only second to malaria as the most devastating parasitic disease in terms of economic and public health impact (Sarvel et al., 2011; Bergquist et al., 2017; World Health Organization, 2019). More than 290 million people are infected worldwide, and an additional 780 million people are at risk for infection (Steinmann et al., 2006; Gryseels, 2012; Beaumier et al., 2013; Global Burden of Disease Study 2013 Collaborators, 2013; Colley et al., 2014; Siddiqui and Siddiqui, 2017).

Praziquantel (PZQ), a drug co-developed by Bayer AG and Merck KGaA in the 1970s, has been implemented in many national programs as an integral component of control and elimination strategies and is the drug of choice for the treatment of schistosomiasis (Olveda et al., 2016; Bergquist et al., 2017; Gower et al., 2017; Kabore et al., 2017; Kimani et al., 2018). Although PZQ is considered non-toxic and highly effective against adult schistosomes, it is unable to kill developing schistosomes and the individual remains susceptible to subsequent infections. Without alternative treatment or control approaches, infection rates in a community can rebound to pretreatment levels when gaps in mass drug treatment occur (Gryseels et al., 1994; N'Goran et al., 2001). Furthermore, the threat of widespread PZQ resistance continues to loom (Ismail et al., 1999; Doenhoff et al., 2008; McManus and Loukas, 2008; Geary et al., 2010; Geary, 2012; Pinto-Almeida et al., 2016). Therefore, the development of an effective vaccine against schistosomiasis is crucial to complement existing control strategies for long-term disease control and, subsequently, elimination.

Typically, vaccines are administered to an individual to generate protective immunity before encountering the targeted pathogen. In the case of schistosomiasis, much of the population in need of receiving a schistosomiasis vaccine has already been previously infected and treated with PZQ (Colley and Secor, 2014; Mo et al., 2014; Siddiqui et al., 2018). It is well-established that helminths modulate the immune response, but little is known about how this modulation affects vaccine efficacy. Evidence suggests that chronic helminth infections during vaccine administration might impair the protective immunity elicited by the vaccine (Gent et al., 2019), especially schistosomiasis which can impair the long-term response to certain vaccines that require predominant Th1 responses (Riner et al., 2016; Wajja et al., 2017). Likewise, PZQ treatment has been shown to alter the polarization of schistosome-specific cytokine responses and could potentially contribute to the development of resistance to reinfection (Bourke et al., 2013). The consensus among experts in the field is that prior to vaccine administration in schistosomiasis-endemic regions, mass PZQ treatment of infected individuals would be a necessity. Hence, the present study was designed to mimic field vaccine deployment conditions in order to gain insight into the cellular immune responses and cytokine expression profiles to schistosome infections, chronic disease progression and PZQ therapy before vaccine administration. We believe that the preliminary data presented here on the cellular immune responses and cytokine expression in baboons during schistosome infection, chronic disease, and following chemotherapy provide additional insight into the immunology of schistosomiasis and could help predict the immune response to further *Schistosoma* antigen exposure in the form of reinfection or a vaccine.

## Materials and Methods

### Statement of Ethics

This study adhered to recommendations for the Care and Use of Laboratory Animals of the National Institutes of Health. Male and female olive baboons (*Papio anubis*) ages 2–6 years old were obtained from the University of Oklahoma Health Sciences Center (OUHSC; Oklahoma City, OK) and housed in Association for Assessment and Accreditation of Laboratory Animal Care-accredited facilities. Baboon studies presented in this publication were approved by the OUHSC IACUC (Protocol Number: 11-160-I). OUHSC maintains a USDA-reviewed program for environmental enhancement to promote psychological well-being of non-human primates. OUHSC is an Assured Institution (Category 1, #A-3165-01) in full compliance with the Public Health Service Policy and approved by the Office for Protection from Research Risks since 1986.

Animals were maintained using social housing practices to ensure regular food and water, sufficient space, and social interactions and stimulation for play. During procedures, baboons were sedated with ketamine 10 mg/kg intramuscular (IM). To minimize distress caused by the isolation during protocol regimens, baboons are returned to the colony/cage-mate as soon as possible or housed separately but adjacent to familiar cohorts.

Infected *Biomphalaria glabrata* (Puerto Rican strain) snails, were obtained from the Schistosomiasis Resource Center, Biomedical Research Institute (Rockville, MD, USA).

### Parasite Challenge, PZQ Therapy, and Isolation of Peripheral Blood Mononuclear Cells

**Figure 1:**
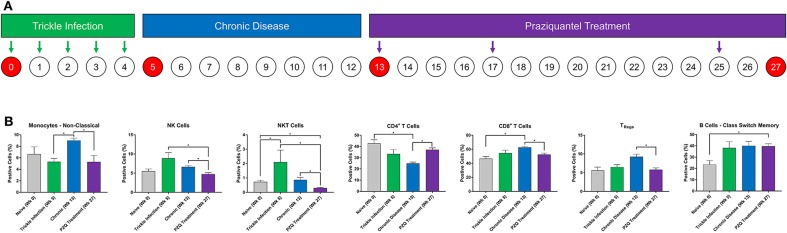
Immune cell lineage analysis during Schistosoma infection and PZQ therapy. Baboons were infected with 200 *S. mansoni* cercariae five times at weekly intervals **(A)**. Peripheral blood (*n* = 4) was collected at the indicated time points and analyzed for changes in myeloid and lymphoid immune cell lineages by flow cytometry **(B)**. Statistical analysis was completed using non-parametric Mann-Whitney *U*-test and findings were determined to be statistically significant at *p < 0.05. Bars represent means with standard error of the mean.

Ten naïve olive baboons were infected 5 times each with 200 *Schistosoma mansoni* cercariae weekly (trickle infection). Development of chronic disease was confirmed by the detection of schistosome eggs in feces. Nine weeks after the final cercarial challenge, all animals were treated three times with PZQ (60 mg/kg oral) at weeks 13, 17, and 25 and monitored weekly for fecal schistosome egg expulsion (Figure 1A). Animals that had no detectable fecal egg output were considered cured. Baboon blood samples were collected at weeks 0, 5, 13, and 25 in heparinized vacutainer tubes (Figure 1A). Peripheral blood mononuclear cells (PBMCs) were isolated from whole blood using Histopaque-1077 (Sigma Aldrich, St. Louis, MO). Purified PBMCs were re-suspended in freezing media (10% DMSO, fetal bovine serum, and RPMI media) for storage at −80°C until further use.

### Flow Cytometry Analysis

Immune cell lineages and functional phenotype were determined by flow cytometry on a BD FACS Melody flow cytometer (BD Biosciences, San Jose, CA), and analyzed using FlowJo v.10 (BD Biosciences, San Jose, CA). Briefly, PBMCs were thawed and viability was assessed using the trypan blue dye exclusion test. PBMCs were stained with the following human antibodies: CD3-BV421 clone: SP34-2, CD4-FITC clone: M-T477, CD8-BV786 clone: RPA-T8, CD14-PE clone: M5E2, CD16-APC clone: 3G8, CD20-FITC clone: 2H7, CD25-PE clone: M-A251, CD27-PE clone: M-T271, CD127-APC clone: H4A3, IgD-APC-H clone: IA6-2 from BD Pharmingen and BioLegend, San Diego, CA. Antibody combinations and gating strategies for specific cell populations are described in Supplementary Table 1 and Supplementary Figure 1. Specific lineage cell populations were gated through either a myeloid or lymphoid cell gate followed by their lineage-specific markers. For the NK cells, we used CD3^−^CD16^+^ because CD56 is not expressed on baboon NK cells until they are expanded *in vitro* (Kennett et al., 2010).

### Cytokine Gene Expression Analysis

Quantitative real-time PCR (qPCR) was carried out to assess the gene expression profile of a panel of Th1, Th2, and Th17 cytokines from the PBMCs obtained at time points discussed above (Figure 1A). Specific primers for qPCR were designed from mRNA sequences obtained from NCBI for *P. anubis* genes. The list of primer sequences used for qPCR is presented in Supplementary Table 2. Briefly, PBMCs were thawed, counted by trypan blue dye exclusion method, and were seeded at 1 × 10^6^ cells/well in a 24-well Costar® plate in complete media (RPMI-1640 supplemented with 10% fetal bovine serum, 100 μg/mL penicillin G, 100 μg/mL streptomycin, and 10 μg/mL gentamycin). Total RNA was extracted from PBMCs using GenElute™ Mammalian Total RNA Miniprep kit (Millipore Sigma, St. Louis, MO) according to manufacturer's instructions. First-strand cDNA synthesis was carried out using Maxima First Strand cDNA synthesis kit (Thermo Fisher Scientific, Waltham, MA) according to manufacturer's protocol. PCR amplification was carried out using SYBR Premix Ex Taq™ (TIi RNase H Plus) (Takara, Japan) on a StepOne™ plus real-time PCR platform (Thermo Fisher Scientific, Waltham, MA) in a reaction volume of 20 μL and primer concentration of 0.2 μM. Reaction conditions were initial denaturation at 95°C for 5 min and then amplification for 40 cycles at 95°C for 5 s, 60°C for 30 s. Glyceraldehyde 3-phosphate dehydrogenase (GAPDH) was used as a housekeeping gene. All reactions were carried out with two technical replicates and ten biological replicates. Relative gene expression was calculated using the 2^−ΔΔ*Ct*^ method with the DataAssist™ software v3.0 (Thermo Fisher Scientific, Waltham, MA) and expressed as Log_2_ (Fold Change).

### Statistical Analysis

Statistical analysis of differences between mean values for proportions of cell groups was determined by non-parametric Mann–Whitney *U*-test. Unpaired parametric two-tailed Student *t*-test was used to analyze cytokines. GraphPad version 8.1.1 software (GraphPad Software Inc., San Diego, CA) was used for all statistical analysis. Bar graph data are presented as mean of biological replicates with standard error of the mean (SEM). Statistical significance was considered based on the criteria that *p* < 0.05. ^*^p < 0.05, ^**^p < 0.01, ^***^p < 0.001, and ^****^p < 0.0001, respectively.

## Results

### Cellular Immune Responses During *Schistosoma mansoni* Infection and PZQ Therapy

Baboons were infected with *S. mansoni* (trickle inoculations), allowed for chronic disease manifestation, and then were treated with praziquantel (Figure 1A). Peripheral blood was collected during each stage of disease development (week 0, 5, and 13) and after completion of PZQ therapy (week 27) and myeloid and lymphoid cell lineages were assessed—these data demonstrate the changes in the cellular immune response following repeated trickle infection, disease progression and subsequent PZQ treatment (Figure 1B). The percentage of myeloid CD14^dim^CD16^+^ non-classical monocytes significantly increased as the disease progressed from week 5 to week 13 into the chronic phase of the disease. Subsequently, the population of non-classical monocytes decreased after PZQ treatment, returning to naïve levels. At week 5, there was an increase in NK cells (*p* = 0.0857) followed by significant decreases following PZQ treatment, returning to naïve levels at week 27. NKT cells demonstrated a similar pattern where the number of cells significantly increased after trickle infection with *S. mansoni* and subsequently dropped to naïve levels after PZQ treatment. We did not observe any significant changes in the levels of classical monocytes but the population of intermediate monocytes had a moderate decrease from trickle infection to PZQ treatment (*p* = 0.0571; Supplementary Figure 2).

Analysis of the lymphoid cells revealed a significant expansion of the CD8^+^ T cell population over the CD4^+^ T cells, peaking during the chronic stage of disease. The ratio of CD4^+^ to CD8^+^ cells shifted from an approximate 1:1 ratio (46.7:42.7, week 0) before infection to a 1:2.5 ratio (24.9:62.9, week 13) during the chronic disease phase. Following PZQ therapy, the ratio of total CD4^+^:CD8^+^ T cells began to revert back toward the levels observed during the trickle infection. To better understand the dynamic changes in the T cell populations during disease progression, we examined the CD4^+^ T-regulatory cell (T_Regs_) populations. We observed an increase in CD3^+^CD4^+^CD25^+^127^−^ T_Regs_ during the chronic phase of the disease (*p* = 0.0571), coinciding with the peak expansion of CD8^+^ T cells, followed by a significant reduction after PZQ treatment. When examining the B cell compartment, the number of CD14^−^CD20^+^IgD^+^CD27^+^ class switch memory B cells significantly increased from week 0 to week 27 with an average from 23.5% (range 13.5–29.6, week 0) to 39.6% (range 35.6–44.5, week 27). We did not observe any significant changes in the levels of naïve B cells nor non-class switch memory B cells throughout this study (Supplementary Figure 2).

### Cytokine Gene Expression Analysis

Cytokines are a category of signaling molecules that mediate and regulate immunity, inflammation and hematopoiesis. The cytokine cascade produced during an immune response determines the type of adaptive immunity developed. During each phase of the experiment, PBMCs were isolated and analyzed for Th1, Th2, and Th17 cytokine gene expression by qPCR to better understand the overall cellular immune response.

**Figure 2:**
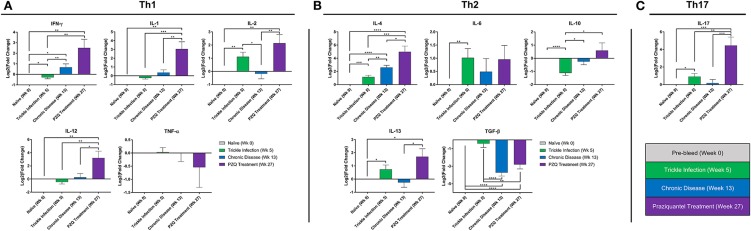
Peripheral blood cytokine expression during Schistosoma infection and PZQ therapy. Quantitative real-time PCR (qPCR) was carried out to assess the expression profile for a panel of Th1, Th2, and Th17 cytokines from the PBMCs obtained at weeks 0, 5, 13, 27 (*n* = 10 for each time point). Bars represent relative fold change expression levels (means + SEM) for **(A)** Th1 cytokines, **(B)** Th2 cytokines, and **(C)** Th17 cytokine expression profiles. *P*-values were calculated using unpaired parametric two-tailed Student *t*-test with *p* < 0.05 considered statistically significant, *p < 0.05, **p < 0.01, ***p < 0.001, and ****p < 0.0001, respectively.

At 5 weeks after the initial *S. mansoni* cercarial infection, we found significant increases in IL-2, IL-4, IL-6, IL-13, and IL-17 gene expression levels and significant decreases in IFN-γ and IL-10 compared to the naïve baboon cytokine levels (Figure 2). Overall, the cytokine expression would suggest by 5 weeks the immune response is polarizing more toward a Th2 type immune response. By week 13, during the chronic phase of the disease, we observed an expected increase in IL-4 as schistosome eggs are being deposited into the liver and intestine. Unexpectedly, by 13 weeks post-initial infection and established chronic disease, we found increased expression in the Th1 cytokine gene IFN-γ compared to naïve and week 5 levels while TGF-β was significantly downregulated. In addition, we found that IL-2, IL-10, IL-13, and IL-17 returned to levels comparable to those of naïve animals at week 13. PZQ treatment stimulated dramatic changes to the cytokine expression profiles, inducing significantly higher expression of almost all cytokines examined: IFN-γ, IL-1, IL-2, IL-12, IL-4, IL-10, IL-13, and IL-17. TGF-β was significantly downregulated, coinciding with the increase in the population of class-switch B memory cells in PBMCs.

## Discussion

Much of our current understanding of the immunology of schistosomiasis is based on murine experimental infection models (Jankovic et al., 1999; Pearce and MacDonald, 2002; Fu et al., 2012; Colley and Secor, 2014; Loc et al., 2019). However, there are intrinsic flaws and limitations associated with these murine models of schistosomiasis (Cheever, 1969; Wilson et al., 2016). Furthermore, widespread use of the most common mouse model, the C57BL/6 mice, in the schistosomiasis vaccine community may obscure the nuances and variations in the immune response that we would observe in populations of an endemic region (Stadecker et al., 2004; Kalantari et al., 2019). Finally, there is a lack of studies that replicate “real world” field conditions, where extensive programs for mass drug administration with PZQ and reinfection are commonplace. In this study, we aimed to address these gaps by utilizing the non-human primate model of schistosomiasis using outbred baboons to study typical conditions in endemic countries, including multiple trickle infection followed by a series of PZQ treatments. Baboons are natural hosts of schistosomes with clinical manifestations similar to those that occur in humans and with a high degree of similarity to human in terms of their immune response (Farah et al., 2001; Siddiqui et al., 2008; Wilson et al., 2016). Herein, we surveyed the changes in the peripheral blood cellular immune response during the establishment of schistosome infection, chronic disease, and PZQ therapy to better understand the state of the immune system that a putative vaccine would encounter in endemic populations.

We observed that a robust cellular immune response developed throughout each phase of our study. From the PBMCs of trickle-infected animals, we found that the population of NKT cells significantly increased compared to naïve animals, coinciding with the increase in the upregulation of IL-2, IL-4, IL-6, IL-13, and IL-17 and a downregulation in IFN-γ, TGF-β, and IL-10. This phase of the immune response is of interest, as the host at this point is responding to different life cycle stages of the parasite; a number of schistosomes from the earliest trickle infection at week 1 may have already developed into adults and have begun to release eggs while schistosomes that have entered the baboons in the latest trickle infection time point may still be in the lungs as schistosomula (Gobert et al., 2007). This may explain the downregulation in IFN-γ at week 5 compared to naïve animals. Increase in IL-2 at week 5 may be correlative to the observed increase in NK cells and CD8^+^ T cells. Although NKT cells have been shown in mice to play a minimal role in the early immune responses to schistosome infection, NKT cells can induce either Th1 or Th2 immune responses at 6 weeks post-infection (Mallevaey et al., 2007).

By week 13, all of the schistosomes would have developed into adults and have laid eggs which would become trapped in tissues or released from the host through feces. It is generally accepted that as schistosomiasis progresses from infection to chronic disease with the onset of egg laying, a Th2 driven response is induced (Pearce and MacDonald, 2002). We observed a significant increase in monocytes and CD8^+^ T cells corresponding with a significant decrease in the number of CD4^+^ T cells. Soluble egg antigens from schistosomes have been demonstrated to be able to stimulate a CD8^+^ T cell response in mice (Pancre et al., 1999). This increase in the number of CD8^+^ T cells, together with the observed increase in IFN-γ may be a response to egg antigens during the chronic phase of schistosomiasis and has been hypothesized to be antifibrinogenic (Pancre et al., 1999; Henri et al., 2002). Furthermore, monocytes have been implicated in the immunopathogenesis of periportal fibrosis (Fernandes et al., 2014) and that intermediate CD14^++^CD16^+^ monocytes have an enhanced ability to bind cercarial and egg excretory/secretory products which may affect an infected individual's ability to respond immunologically to infection (Turner et al., 2014). NK cells and NKT cells reverted back to naïve levels as the immune response shifted form innate immunity toward adaptive immunity. When we measured the cytokine expression at week 13, we observed a significant increase in IFN-γ and IL-4 and a significant decrease in IL-2 and TFG-β. Relative to week 5, the level of IL-10 increased, suggesting that immunomodulation is occurring to reduce inflammatory responses to eggs, coinciding with an increase in the number of T_regs_ (*p* = 0.0571) at week 13. Th2 responses also facilitate B cell isotype switching. From the naïve state to the chronic state, we found that class-switch IgD^−^CD27^+^ memory B cells increased from 23.5 % (range 13.5–29.6, week 0) to 40.1 % (range 29.1–47, week 13) (*p* = 0.0571); whereas the non-class-switch IgD^+^CD27^+^ memory B cell population remained relatively unchanged (Supplementary Figure 2).

Following PZQ therapy, most of the cell populations measured from PBMCs collected at week 27 reverted back toward near naïve levels with the exception of B cell populations overall which remained elevated with an increased class-switch memory phenotype. The overall observed cellular immune responses are in-line with many other reports where a predominantly T-helper 1 reaction in the early stages of infection shifts to an egg-induced Th2 biased profile (Pearce and MacDonald, 2002; Colley and Secor, 2014). However, the cytokine expression profile of PBMCs following PZQ therapy shows a mixed Th1/Th2/Th17 response with nearly all of the cytokines measured were upregulated compared to the cytokine profile at week 13 with the exceptions of TGF-β being downregulated and TNF-α and IL-6 showing no significant change. Destruction of the adult schistosome worm releases antigens that may be concealed from the host immune response, such as antigens in the tegument, thus causing a strong cellular immune response, although marked variability of cytokine expression between individuals can exist (Joseph et al., 2004a; Martins-Leite et al., 2008; Castro et al., 2018; Supplementary Figure 3).

It is a common knowledge that repeated infection with *Schistosoma* generates an adaptive immune response(s) and eventually provides some resistance to reinfection, although it may take a long time and rarely results in sterile immunity (Mutapi et al., 1999; Fitzsimmons et al., 2012; Colley and Secor, 2014). A vaccine that can drive immune responses toward protective immunity and reduce pathology is strongly needed. Yet, models that replicate “real world” field conditions to effectively evaluate putative schistosomiasis vaccines are lacking. Conditions such as HIV (Joseph et al., 2004b), coinfection with whipworm (our unpublished data show increased *Schistosoma* egg-induced hepatopathology during co-infection with *Trichuris*), malnutrition (Scrimshaw and SanGiovanni, 1997), and others can affect vaccine efficacy. PZQ treatment, discounting the release of parasite antigens, may intrinsically be immunomodulatory (Eyoh et al., 2019). To our knowledge, this study is the first to report the cellular immune response in a non-human primate model of schistosomiasis using multiple trickle infections and subsequent chemotherapy with PZQ. Due to the logistical problems and high cost associated with the non-human primate model, there are inherent limitations and less flexibility as it relates to the experimental design. Therefore we are limited to a small number of strategies that can be tested in baboons. However, we plan to perform additional studies that may include expansion of sample size, cell sorting to correlate specific cytokine responses to specific cell types, and functional assays to further delineate the nuanced cellular immune response to schistosomiasis followed by chemotherapy with praziquantel. Overall, the data presented in this study may be important for developing potential markers and/or correlates of protection to monitor and evaluate putative vaccines for acquired immunity.

## Data Availability Statement

All datasets generated for this study are included in the article/Supplementary Material.

## Ethics Statement

This animal study was reviewed and approved by Oklahoma University Health Sciences Center IACUC (Protocol No. 11-160-I).

## Author Contributions

AAS conceived and designed the study. MM and AJS performed the flow cytometry. AJS and SL performed the real time PCR. LL analyzed the data and created the figures. MM and LL wrote the manuscript. AM and WZ wrote sections of the manuscript. All authors contributed to manuscript revision and approved the submitted version.

### Conflict of Interest

The authors declare that the research was conducted in the absence of any commercial or financial relationships that could be construed as a potential conflict of interest.
