# Supplementary material for: Elucidation of Cellular Responses in Non-human Primates With Chronic Schistosomiasis Followed by Praziquantel Treatment
Source: Front Cell Infect Microbiol. 2020 Feb 24;10:57. doi: 10.3389/fcimb.2020.00057 (PMC7050631; doi:10.3389/fcimb.2020.00057)
Supplement: Supplementary file 1 [file Data_Sheet_1.docx]

**Elucidation of Cellular Responses in Nonhuman Primates with Chronic Schistosomiasis followed by Praziquantel Treatment**

**Supplementary Materials**

**Supplementary Table 1. Antibody combinations for flow cytometry immune cell phenotyping.**

| **Antibody Combination** | **Lineage Cell Type** | **Phenotype** |
| --- | --- | --- |
| CD3-BV421 | T cell | CD3+ |
| CD20-FITC | B cell | CD20+ |
| CD16-APC | NK cell | CD3- CD16+ |
| CD14-PE | NKT cell | CD3+ CD16+ |
|  | Monocyte/Macrophage | CD14+ CD3- CD20- |
|  | Monocyte Classical | CD14++ CD16- |
|  | Monocyte Intermediate | CD14++ CD16+ |
|  | Monocyte Non-classical | CD14dim CD16+ |
| **Antibody Combination** | **T Cell Type** | **Phenotype** |
| CD3-BV421 | T cell | CD3+ |
| CD4-FITC | CD4 T cell | CD3+ CD4+ CD8- |
| CD8-BV786 | CD8 T cell | CD3+ CD8+ CD4- |
| CD25-PE | T Regulatory cell | CD3+ CD4+ CD25+ CD127- |
| CD127-APC |  | |
| **Antibody Combination** | **B Cell Memory** | **Phenotype** |
| CD20-FITC | B cell | CD20+ |
| CD27-PE | Naïve | CD20+ IgD+ CD27- |
| IgD-APC-H7 | Non-class Switch Memory | CD20+ IgD+ CD27+ |
|  | Class Switch Memory | CD20+ IgD- CD27+ |

**Supplementary Table 2. Cytokine primer sequences.**

| **Gene** | | **Primer sequence** |
| --- | --- | --- |
| ***GAPDH*** | Sense | 5′-AACATCATCCCTGCCTCTACTG-3′ |
|  | Antisense | 5′-TTGGCAGGTTTTTCCAGACG-3′ |
| ***IFN-γ*** | Sense | 5′-TGTGGAGACCATCAAGGAAGAC-3′ |
|  | Antisense | 5′-TGTACTGCTTTGCGTTGGAC-3′ |
| ***TNF-α*** | Sense | 5′-AGCCCATGTTGTAGCAAACC-3′ |
|  | Antisense | 5′-ATGAGGTACAGGCCTTCTGAT-3′ |
| ***IL-2*** | Sense | 5′-TGTACAGGATGCAACTCCTGTC-3′ |
|  | Antisense | 5′-GCTCCAGTTGTAGCTGTGTTTT-3′ |
| ***IL-12α*** | Sense | 5′-ACCAGGTGGAGTTCAAGACCA-3′ |
|  | Antisense | 5′-GCCCGAATTCTGAAAGCATG-3′ |
| ***IL-1α*** | Sense | 5′-ATGAAGACCAACCAGTGCTG-3′ |
|  | Antisense | 5′TTGGATGGGCAACTGATGTG 3′ |
| ***IL-17*** | Sense | 5′-CAATCCCACGAAATCCAGGATG-3′ |
|  | Antisense | 5′-GGTGGAGATTCCAAGGTGAGG-3′ |
| ***IL-4*** | Sense | 5′-TGCCTCCAAGAACACAACTG-3′ |
|  | Antisense | 5′-AATCGGATCAGCTGCTTGTG-3′ |
| ***IL-10*** | Sense | 5′-ACATCAAGGCGCATGTGAAC-3′ |
|  | Antisense | 5′-ACGGCCTTGCTCTTGTTTTC-3′ |
| ***IL-13*** | Sense | 5′-AGTTTTCCAGCTTGCGTGTC-3′ |
|  | Antisense | 5′-AACTGTCCCTCGCGAAAAAG-3′ |
| ***TGF-β*** | Sense | 5′-TTGATGTCACCGGAGTTGTG-3′ |
|  | Antisense | 5′-TGATGTCCACTTGCAGTGTG-3′ |
| ***IL-6*** | Sense | 5′-TGCAATAACCACCCCTGAAC-3′ |
|  | Antisense | 5′-TTAAAGCTGCGCAGGATGAG-3′ |

**
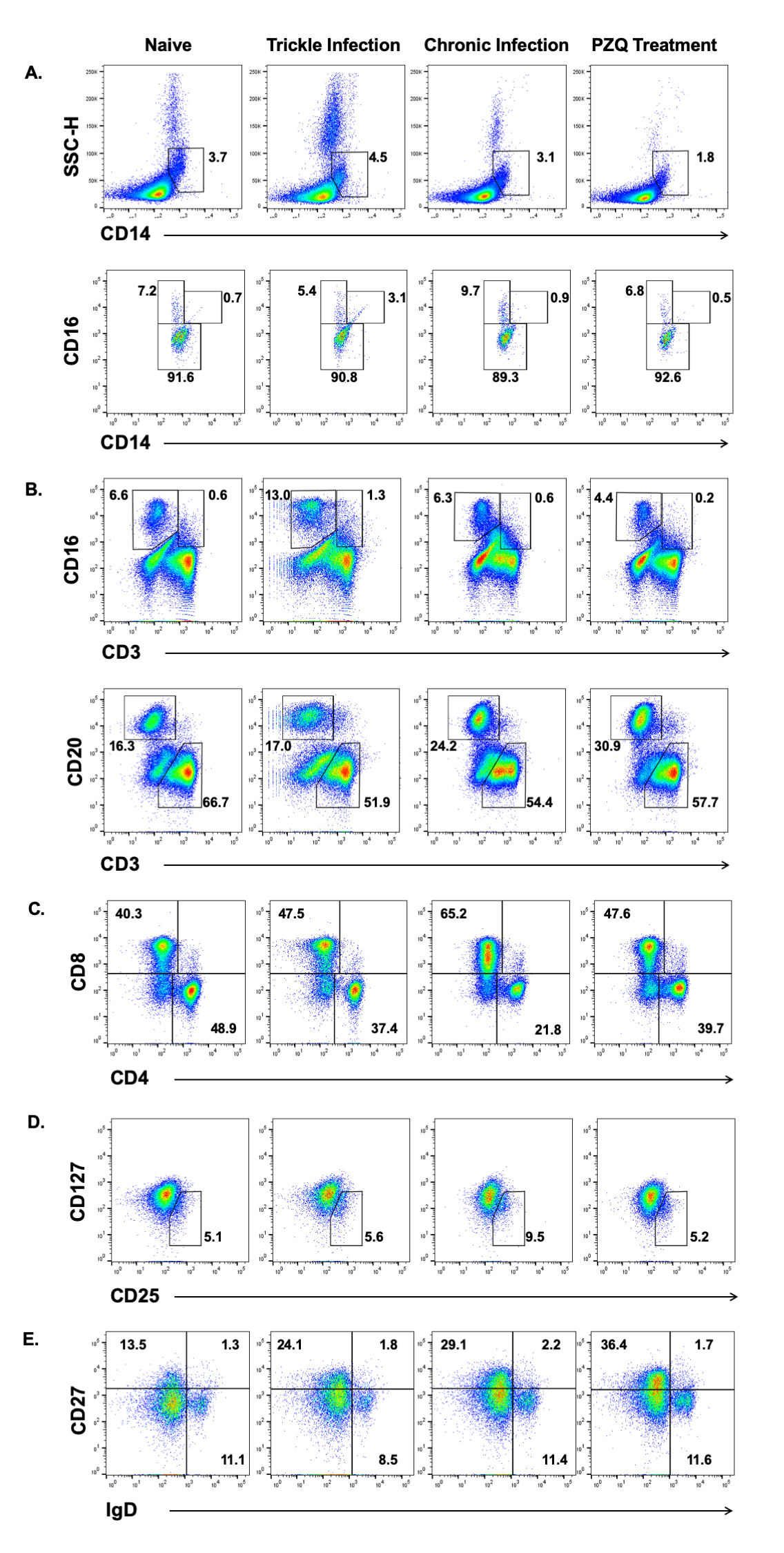
**

**Supplementary Figure 1. Representative flow cytometry gating strategies.** Cells were gated for single cells based on Forward Scatter-Height versus Forward Scatter-Area. The single cells were then gated to eliminate debris using Forward versus Side Scatter (our live cell gate). Lineage cell populations were then gated through the Live Cell Gate and their lineage-specific markers (Supplementary Table 1). Panel A depicts the gating strategy for monocytes, Panel B depicts the gating strategy for NK cells, NKT cells, T cells, and B cells. Panel C depicts the gating strategy for CD4^+^ and CD8^+^ T cells. Panel D depicts the gating strategy for T_regs_. Panel E depicts the gating strategy for memory B cells. All representative gating strategies are shown using samples from Animal 1.

**
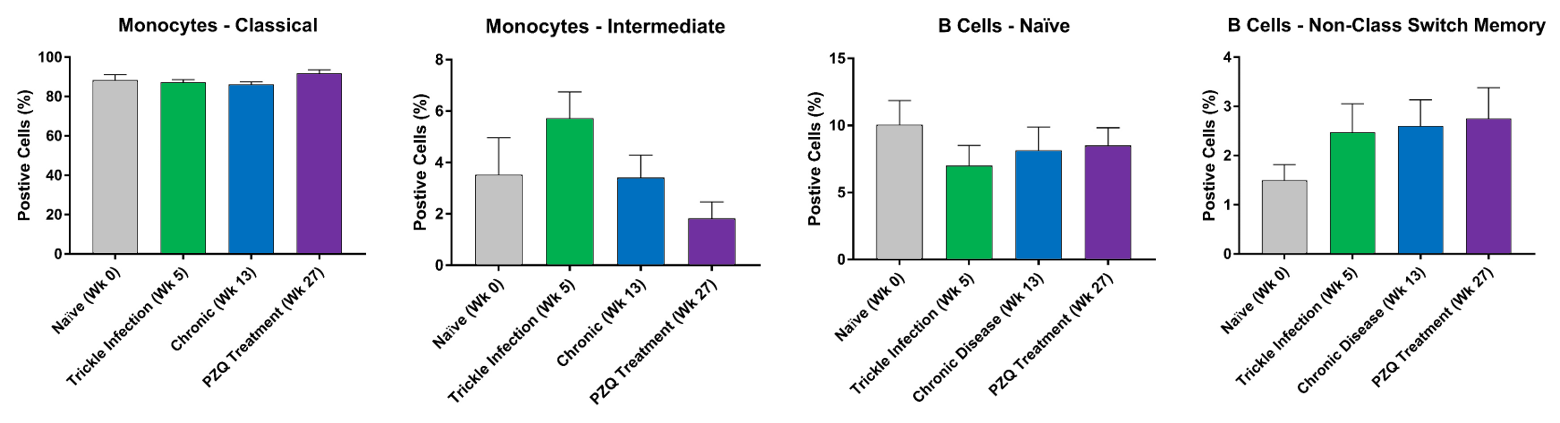
**

**Supplementary Figure 2. Flow cytometry.** Peripheral blood (*n* = 4) was collected at weeks 0, 5, 13, and 27 and subsequently analyzed for changes in myeloid and lymphoid immune cell lineages by flow cytometry. Statistical analysis was completed using nonparametric Mann-Whitney *U*-test and findings were determined to be statistically significant at *p* < *0.05. Bars represent means with standard error of the mean.

**
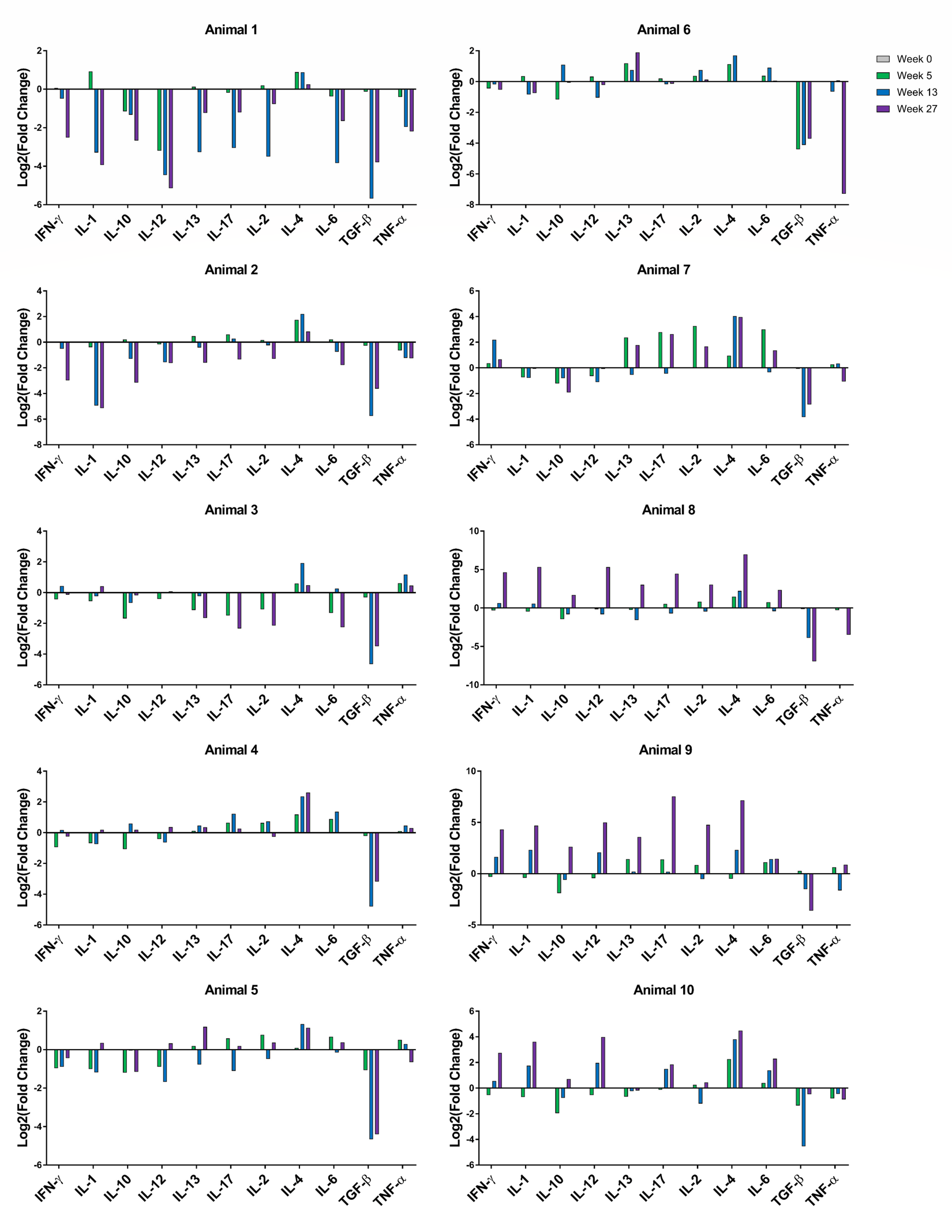
**

**Supplementary Figure 3. Cytokine profiles from individual baboons exhibit variability in immune responses to schistosome trickle infection and PZQ treatment.** Quantitative real‐time PCR (qPCR) was carried out to assess the expression profile for a panel of Th1, Th2, and Th17 cytokines from the PBMCs obtained at weeks 0, 5, 13, 27 (*n* = 10 for each time point). Bars represent relative fold change expression levels (means of 2 replicates per cytokine for each timepoint).
